# CAG Student Prize Paper – A5 NON-INVASIVE PEPTIDOMIC SIGNATURE DISTINGUISHING ACTIVE AND REMISSIVE IBD USING A NESTED CROSS-VALIDATED MACHINE-LEARNING STUDY

**DOI:** 10.1093/jcag/gwaf042.005

**Published:** 2026-02-13

**Authors:** E Shajari, D Gagné, M Malick, P Roy, M Delisle, M Brunet, F Boisvert, J Beaulieu

**Affiliations:** Immunology and Cell Biology, Universite de Sherbrooke Faculte de Medecine et des Sciences de la Sante, Sherbrooke, QC, Canada; Immunology and Cell Biology, Universite de Sherbrooke Faculte de Medecine et des Sciences de la Sante, Sherbrooke, QC, Canada; Immunology and Cell Biology, Universite de Sherbrooke Faculte de Medecine et des Sciences de la Sante, Sherbrooke, QC, Canada; Immunology and Cell Biology, Universite de Sherbrooke Faculte de Medecine et des Sciences de la Sante, Sherbrooke, QC, Canada; Immunology and Cell Biology, Universite de Sherbrooke Faculte de Medecine et des Sciences de la Sante, Sherbrooke, QC, Canada; Immunology and Cell Biology, Universite de Sherbrooke Faculte de Medecine et des Sciences de la Sante, Sherbrooke, QC, Canada; Immunology and Cell Biology, Universite de Sherbrooke Faculte de Medecine et des Sciences de la Sante, Sherbrooke, QC, Canada; Immunology and Cell Biology, Universite de Sherbrooke Faculte de Medecine et des Sciences de la Sante, Sherbrooke, QC, Canada

## Abstract

**Background:**

Monitoring disease activity in inflammatory bowel disease (IBD), encompassing Crohn’s disease and ulcerative colitis, is critical for guiding therapy and preventing irreversible mucosal damage. Colonoscopy, the current gold standard, is invasive and impractical for frequent follow-up, while fecal calprotectin lacks precision within its diagnostic “gray zone.” In this context, stool proteomics provides a non-invasive window into intestinal inflammation through direct measurement of molecular effectors.

**Aims:**

To establish a proof-of-concept study demonstrating that stool-derived peptides can be leveraged for accurate IBD activity classification (Active vs Remission) using an unbiased, reproducible nested cross-validation (NCV) machine-learning approach.

**Methods:**

A total of 170 stool samples from IBD patients were collected and profiled using SWATH-DIA mass spectrometry. Feature selection was performed within the training loops only (Boruta, LASSO, RRF) across repeated subsampling, retaining peptides consistently identified in ≥ 70 % of runs. Stable features were used to train four classifiers (GLMNet, SVM-Radial, SVM-Linear, Naïve Bayes) under inner 5-fold tuning. Outer test folds provided fully unseen evaluation, and misclassified cases were tracked to assess biological ambiguity.

**Results:**

Feature selection yielded 8–12 stable peptides per fold, mapping to 9 unique proteins, several of which recurred across ≥60% of outer folds. Models showed high and stable performance across folds with AUC = 0.94–0.97 and Balanced Accuracy = 0.84–0.90. Specificity remained high (≥ 0.90), while sensitivity ranged 0.77–0.93, confirming reliable detection of both active and remission states. Inner-CV results highlighted GLMNet and SVM-Radial as consistently strong performers, whereas Naïve Bayes achieved slightly higher mean sensitivity. Only a few borderline samples were repeatedly misclassified, suggesting biological rather than algorithmic uncertainty.

**Conclusions:**

This work provides a proof of concept that stool-based peptidomic features can serve as reliable indicators of IBD activity when analyzed through robust, leakage-free machine-learning design. The study establishes a foundation for future translational studies aiming to refine and clinically implement stool peptide biomarkers as part of personalized, non-invasive IBD management.

**Funding Agencies:**

CCC, CIHR

